# Supplementary figures and images for: Aging decreases docosahexaenoic acid transport across the blood-brain barrier in C57BL/6J mice
Source: PLoS One. 2023 Feb 16;18(2):e0281946. doi: 10.1371/journal.pone.0281946 (PMC9934487; doi:10.1371/journal.pone.0281946)

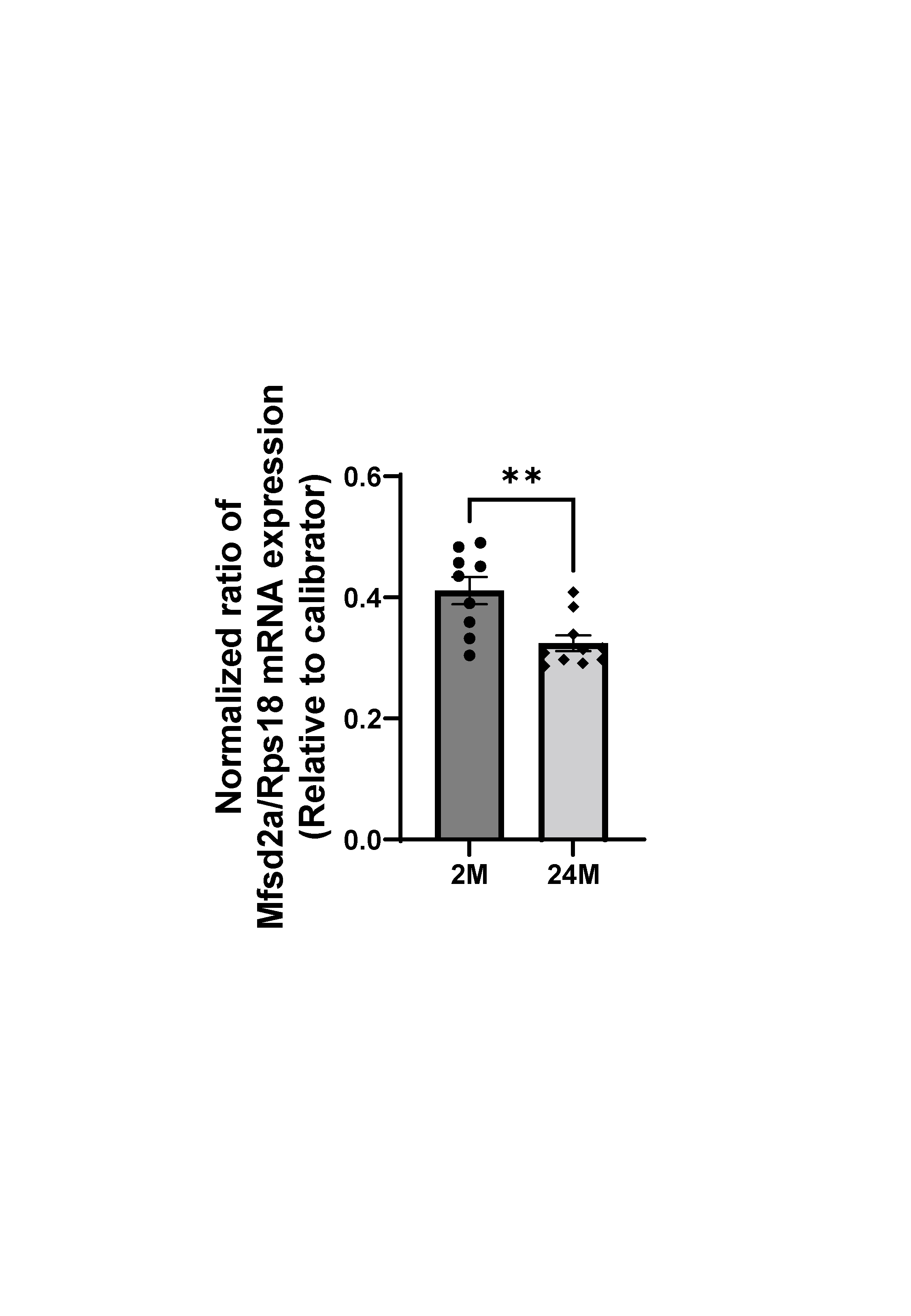

Supplement: S1 Fig — The mRNA expression levels for Mfsd2a in brain microvessels from 2- (2M) and 24-month-old (24M) mice were quantified by real-time quantitative PCR. Data are shown as the mean ± standard error of the mean. Each closed symbol represents an individual value (n = 9–10). **P < 0.01, significantly different from 2-month-old group. (TIFF) [file pone.0281946.s001.tiff]
