# Supplementary material for: Aging decreases docosahexaenoic acid transport across the blood-brain barrier in C57BL/6J mice
Source: PLoS One. 2023 Feb 16;18(2):e0281946. doi: 10.1371/journal.pone.0281946 (PMC9934487; doi:10.1371/journal.pone.0281946)
Supplement: S1 File — (DOCX) [file pone.0281946.s002.docx]

**Supplementary method**

**Real-time quantitative PCR**

　　　　The method of Real-time quantitative PCR is previously described (1). PCR was performed through 45 cycles of 95°C for 10 seconds, 60°C for 20 seconds, and 72°C for 1 minute, using specific primers purchased from Takara Bio Inc (Shiga, Japan). The primer sequences are shown as follows: sense primer 5′- TTCTGGCCAACGGTCTAGACAAC -3’, and antisense primer 5′- CCAGTGGTCTTGGTGTGCTGA -3’ for Rps18 (NM_011296.3); sense primer 5′- TGGCCCTCATGGAGCGTAA -3’, and antisense primer 5′- CATCGATAACGTCAGGCAGCA -3’ for Mfsd2a (NM_029662.2). The gene of Rps18 was used as a house keeping gene. Threshold cycle values (Cq) of each sample were normalized by Cq of calibrator.

**Reference**

1. Takata F, Dohgu S, Matsumoto J, Machida T, Sakaguchi S, Kimura I, et al. Oncostatin M-induced blood-brain barrier impairment is due to prolonged activation of STAT3 signaling in vitro. J Cell Biochem. 2018;119(11):9055-63.
