# Supplementary material for: Aging decreases docosahexaenoic acid transport across the blood-brain barrier in C57BL/6J mice
Source: PLoS One. 2023 Feb 16;18(2):e0281946. doi: 10.1371/journal.pone.0281946 (PMC9934487; doi:10.1371/journal.pone.0281946)

S1_raw_images (Fig. 3)

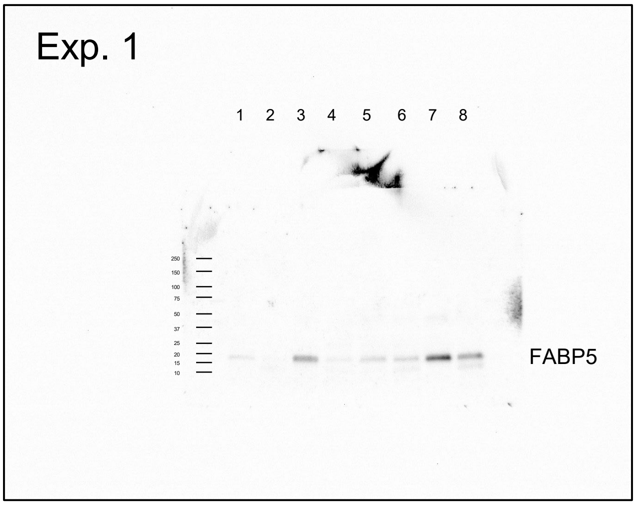

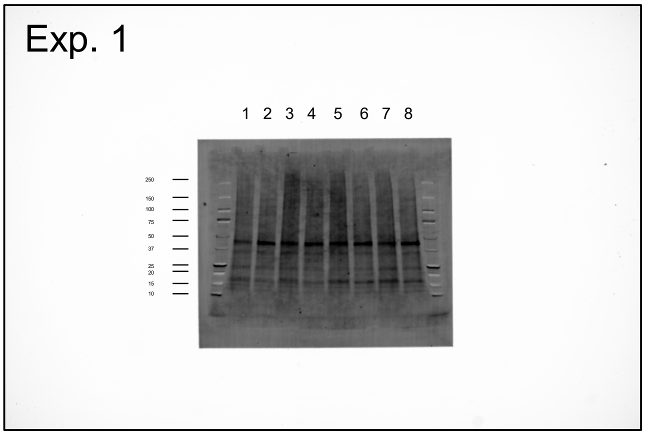

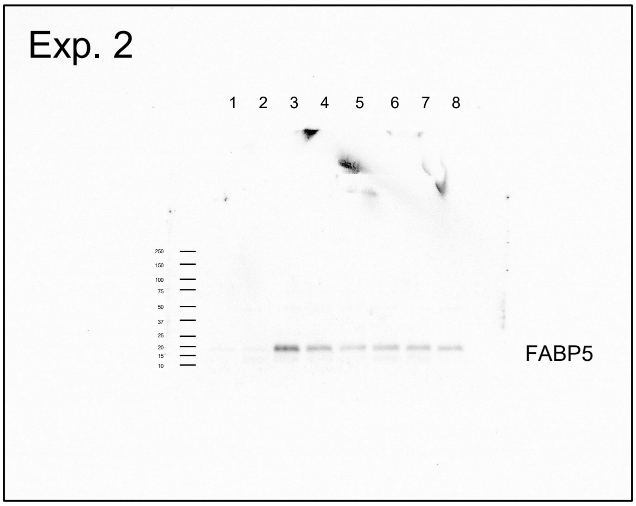

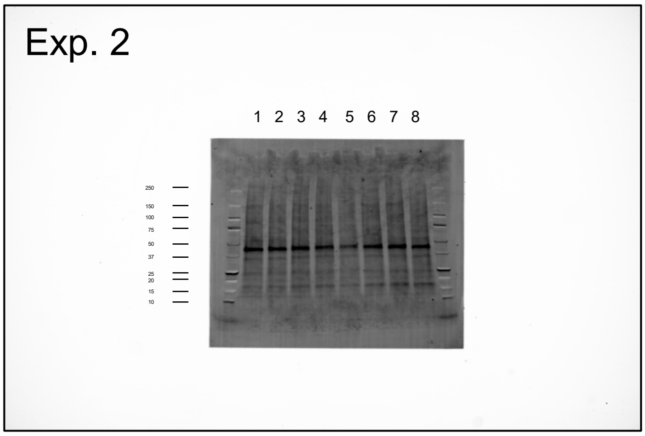

Used in Fig. 3

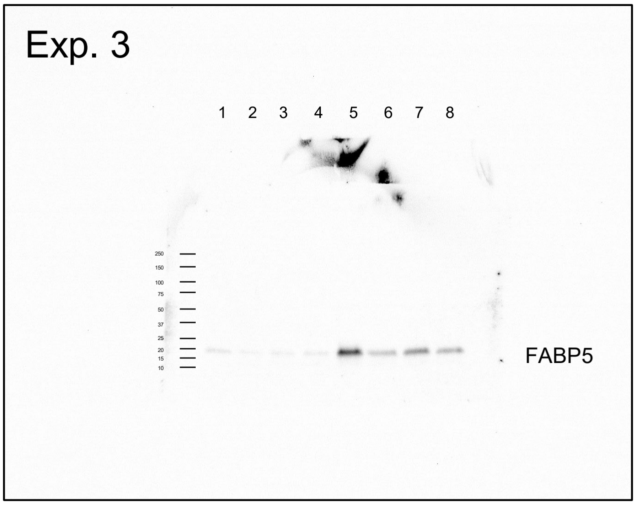

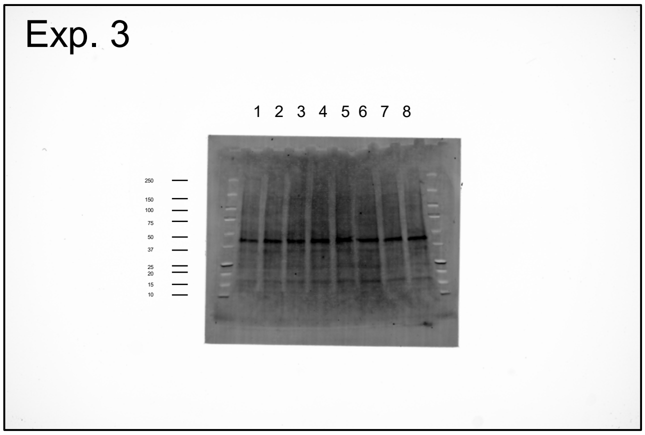

FABP5

Total protein (loading control)

Lane 1	2-month-old mice 1
Lane 2	2-month-old mice 2
Lane 3	8-month-old mice 1
Lane 4	8-month-old mice 2
Lane 5	12-month-old mice 1
Lane 6	12-month-old mice 2
Lane 7	24-month-old mice 1
Lane 8	24-month-old mice 2

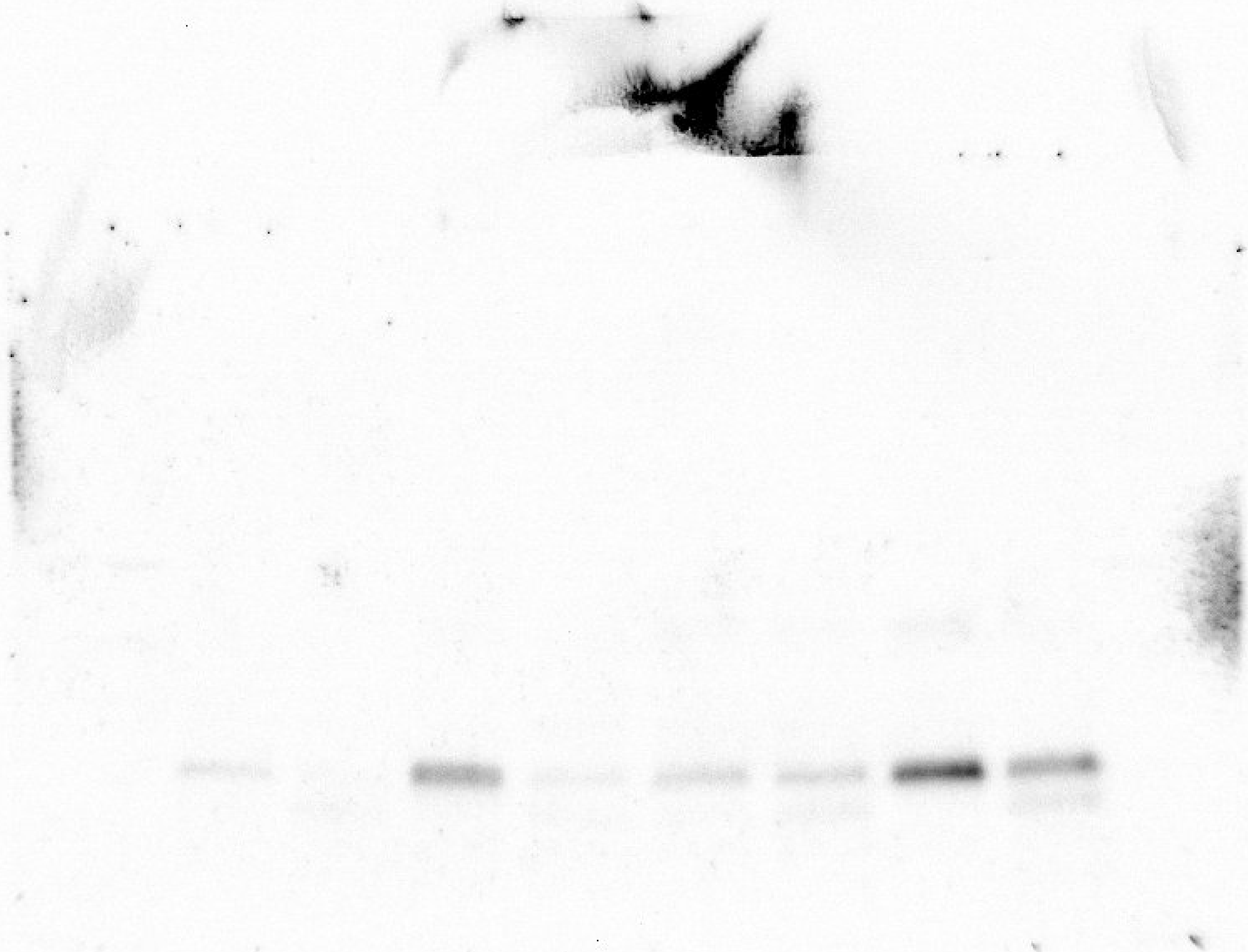

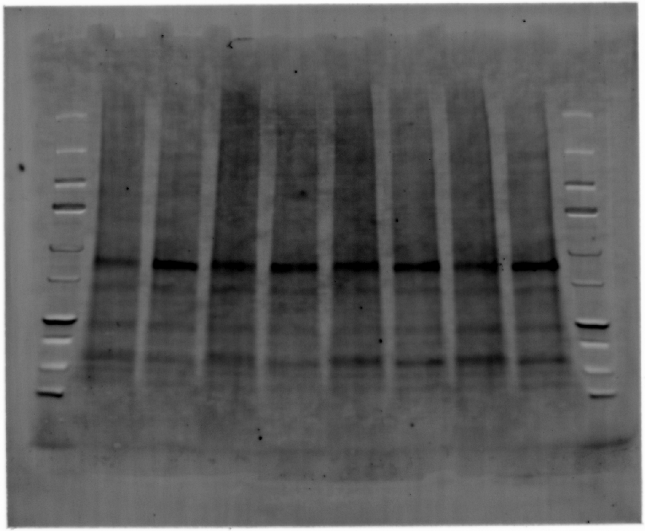

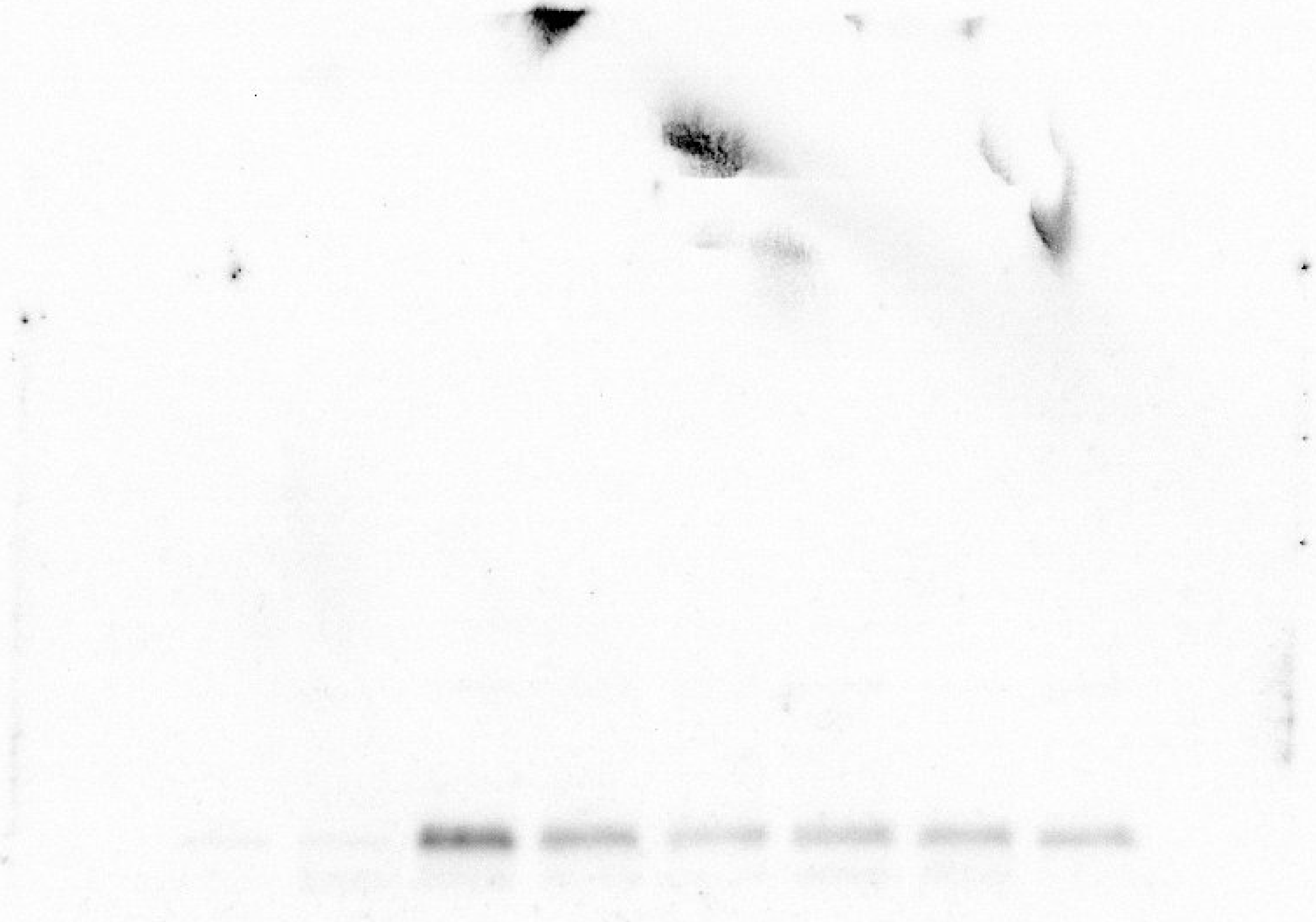

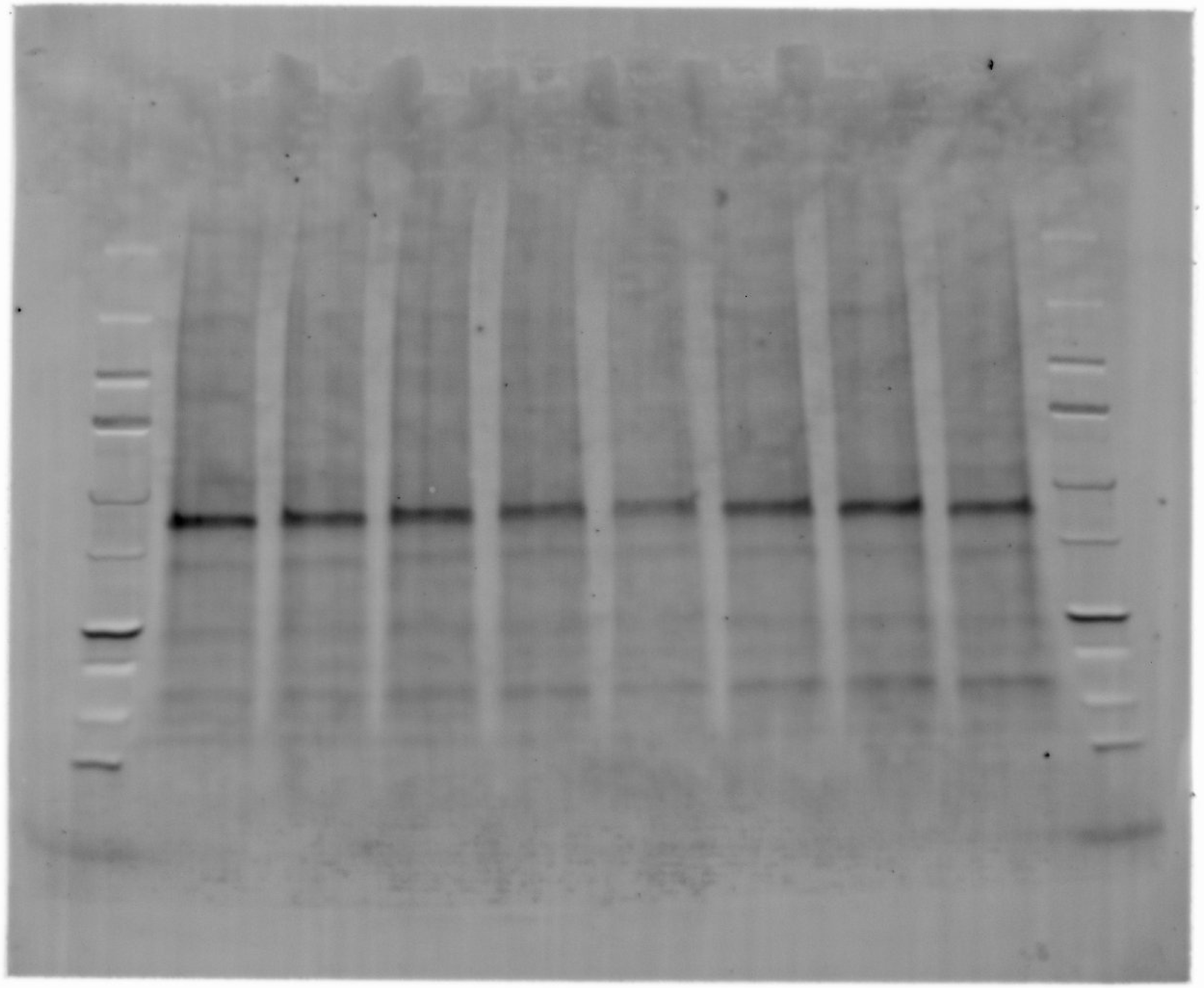

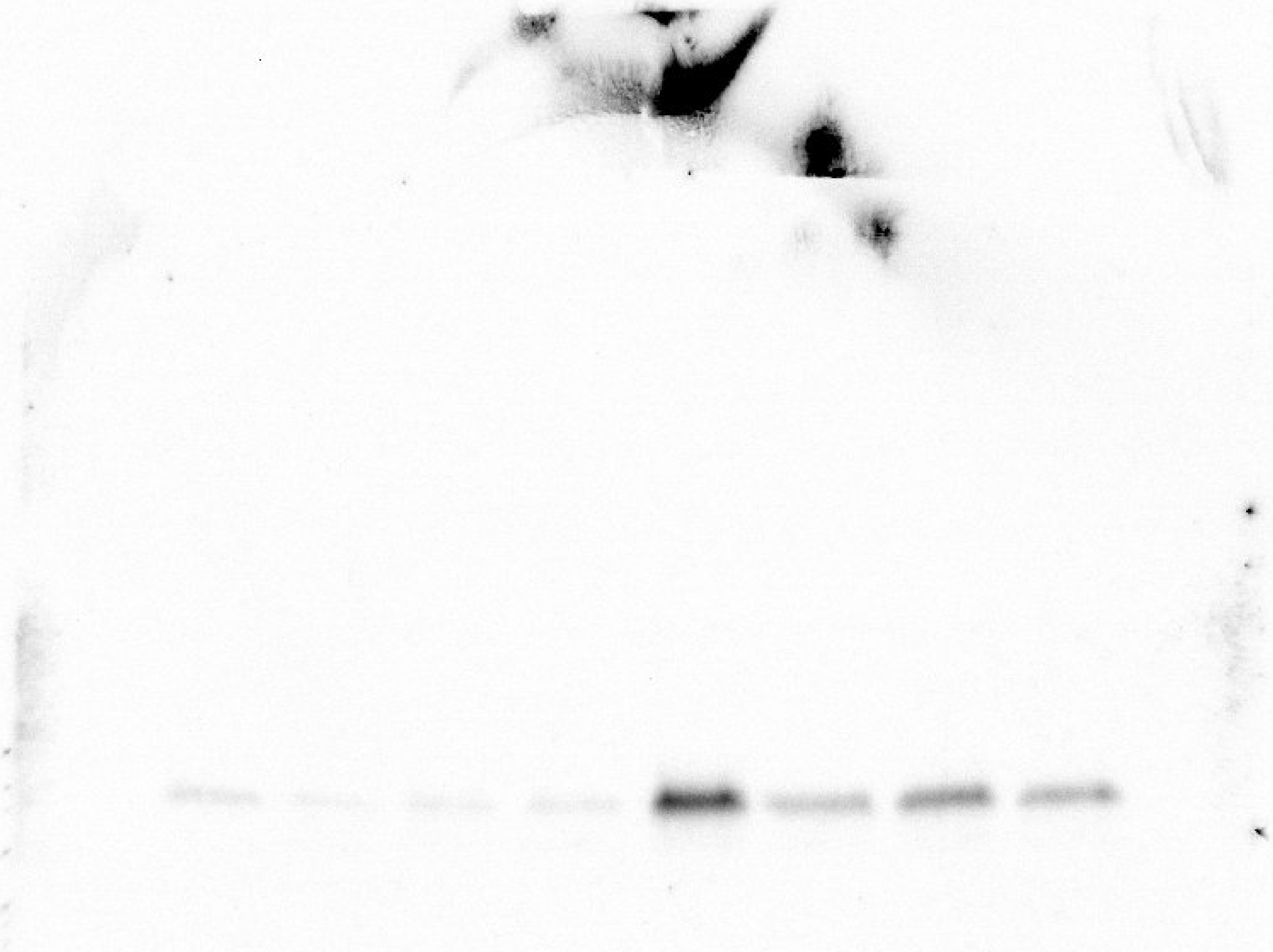

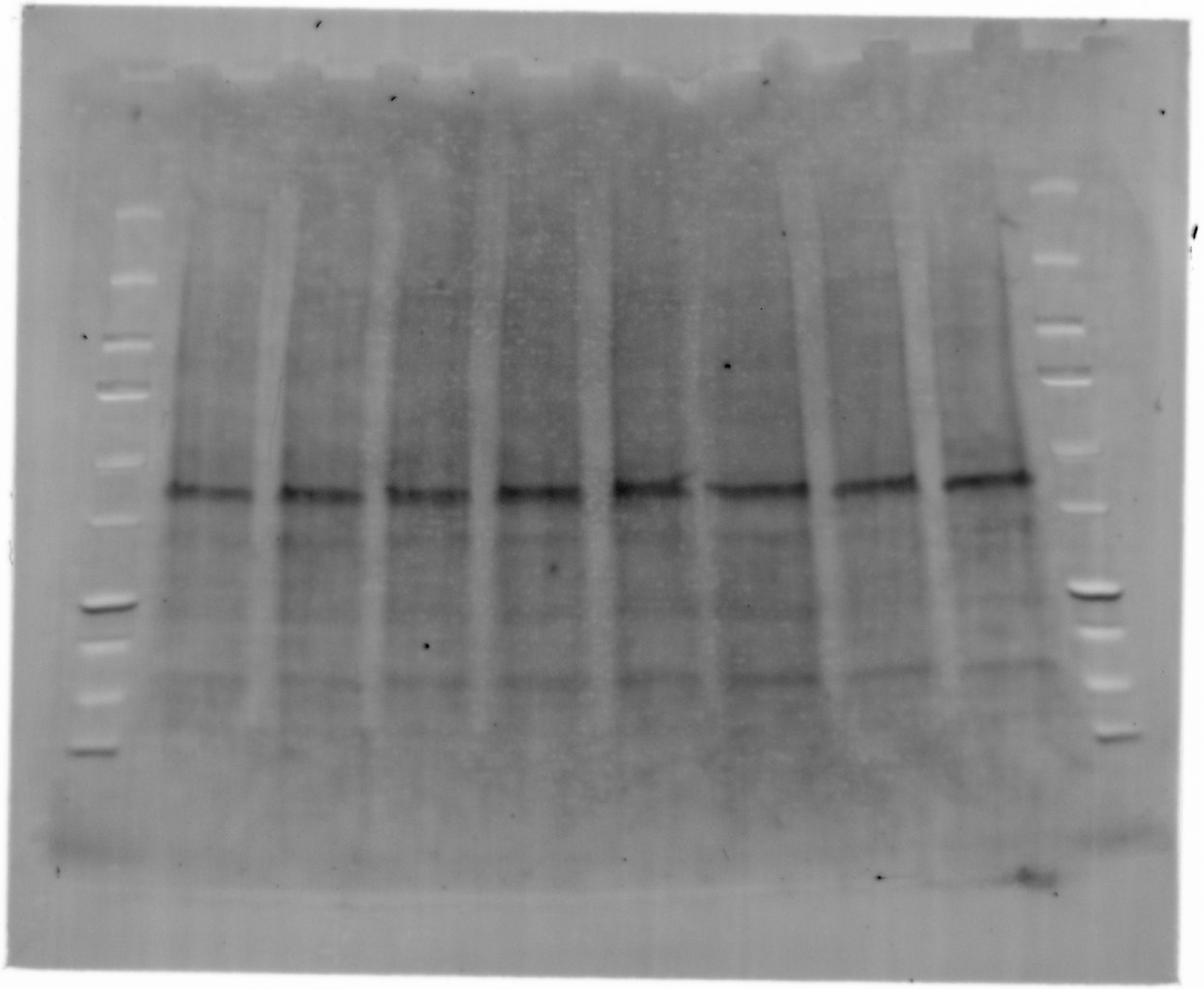

S1_raw_images (Fig. 6)

Used in Fig. 6

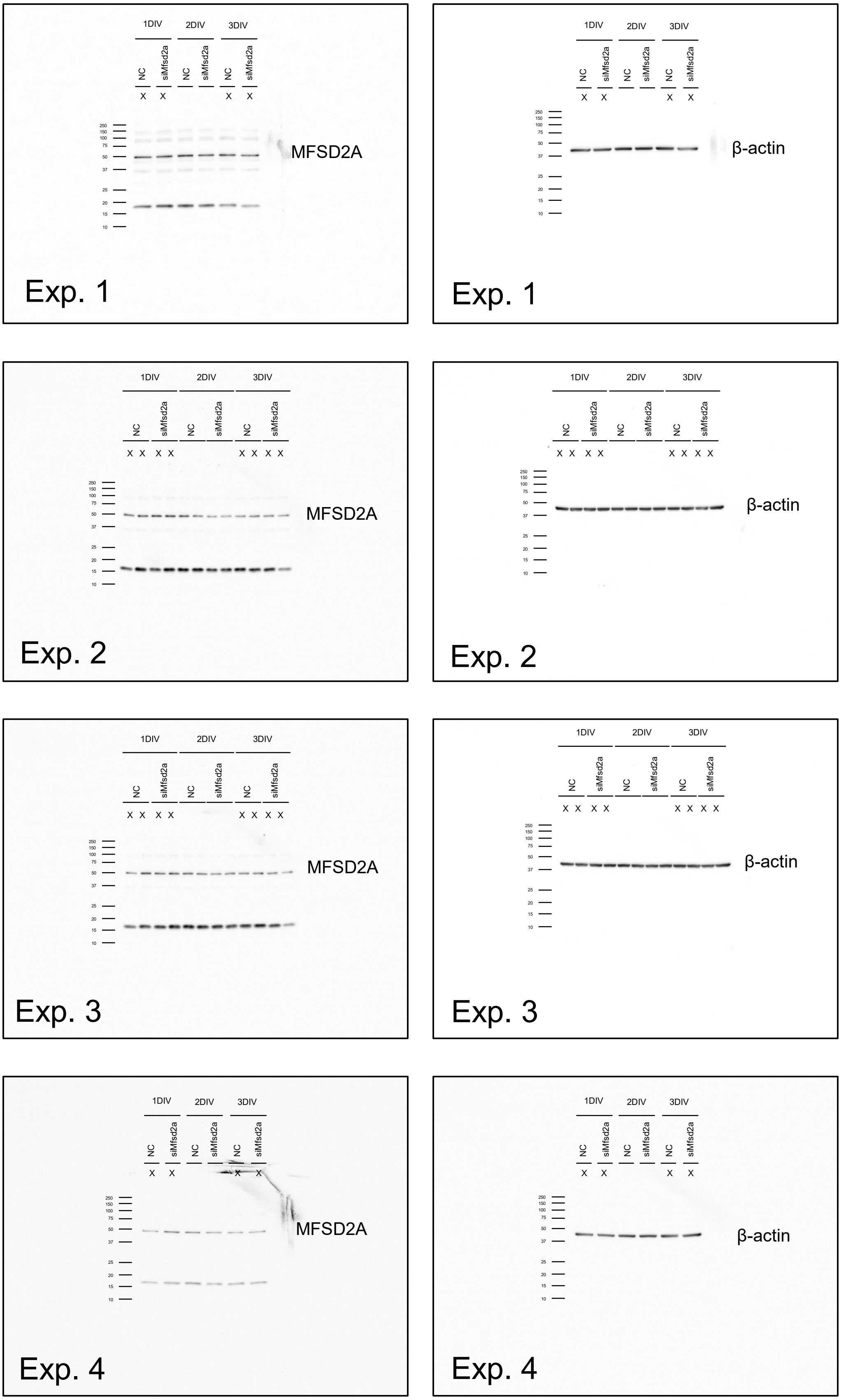

MFSD2A

β-actin (loading control)

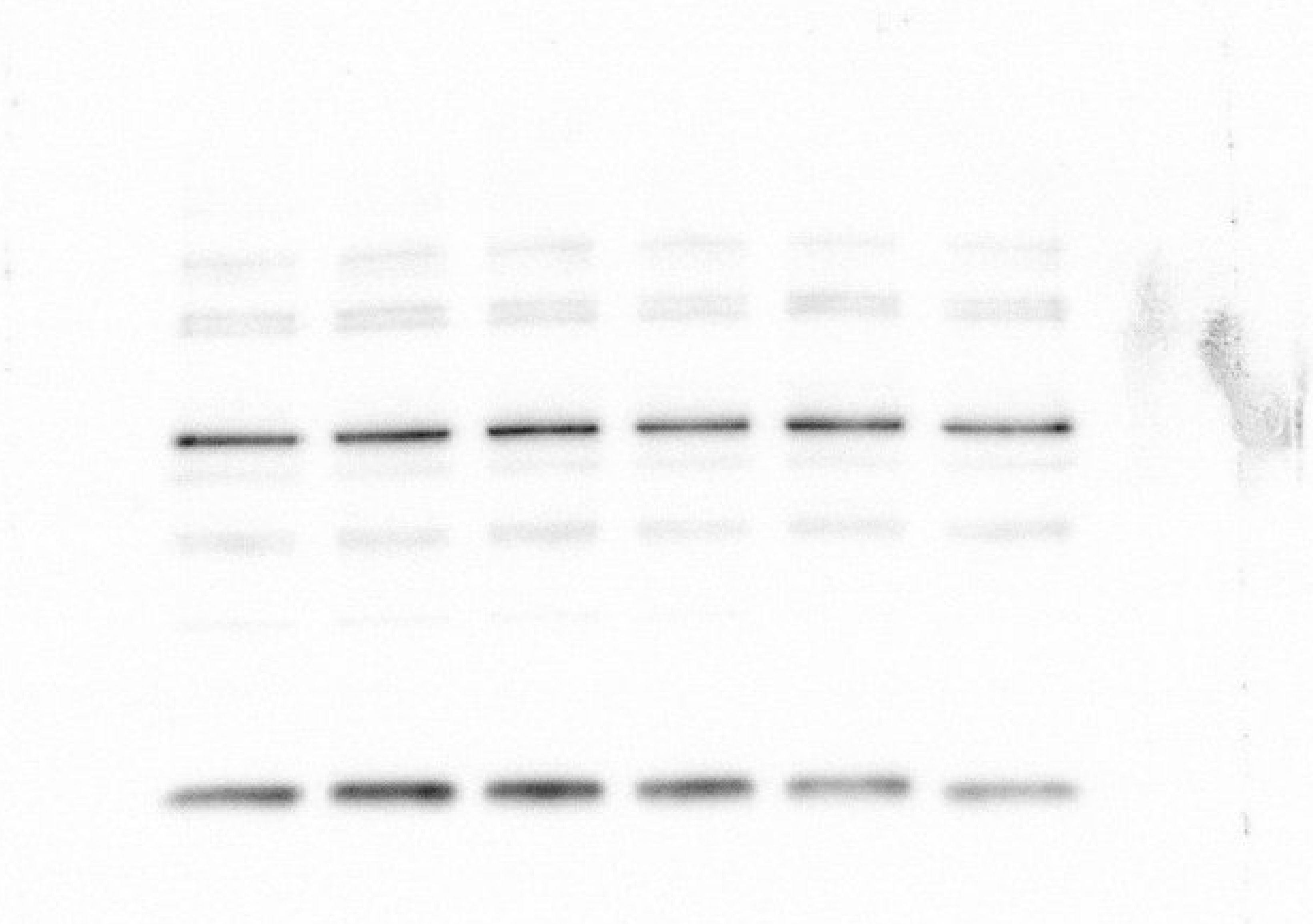

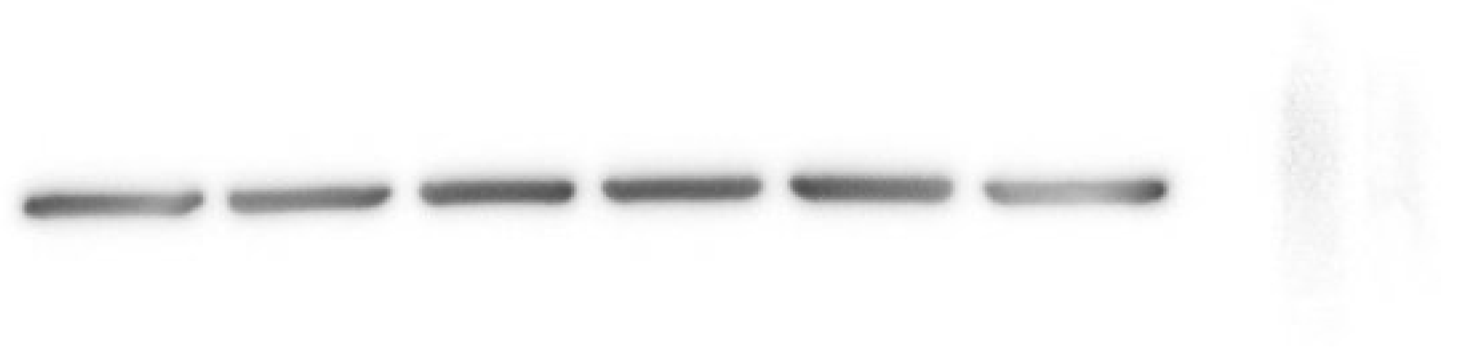

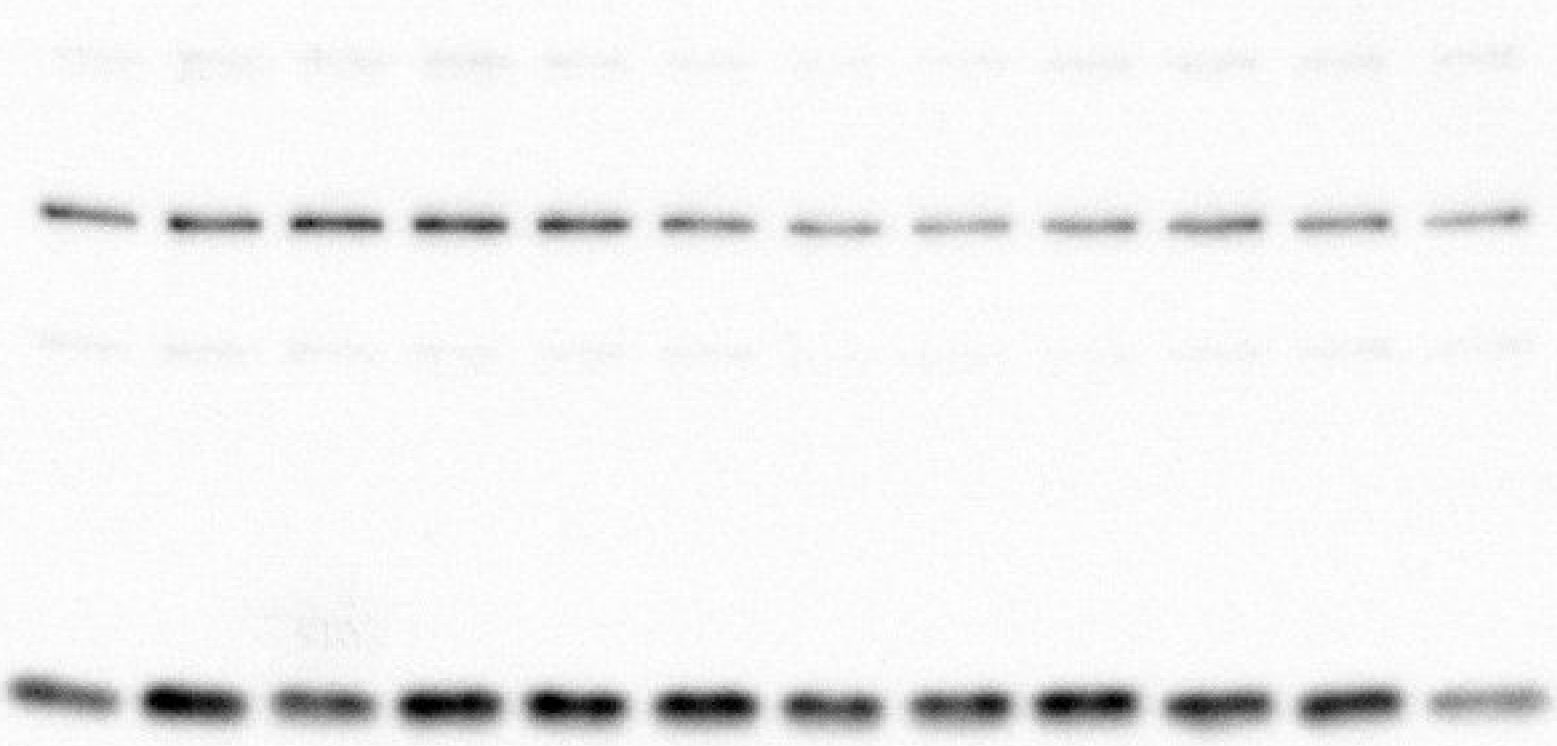

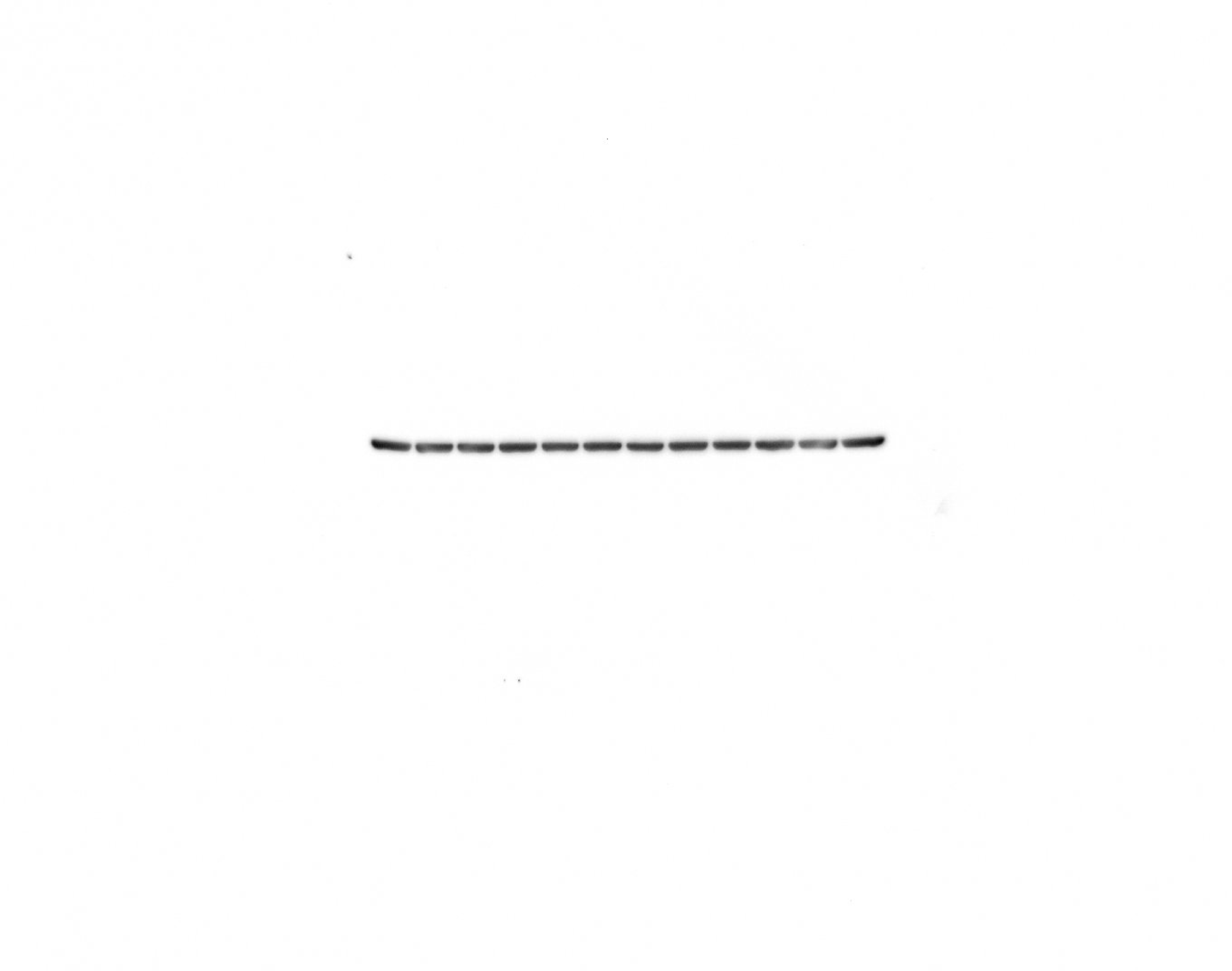

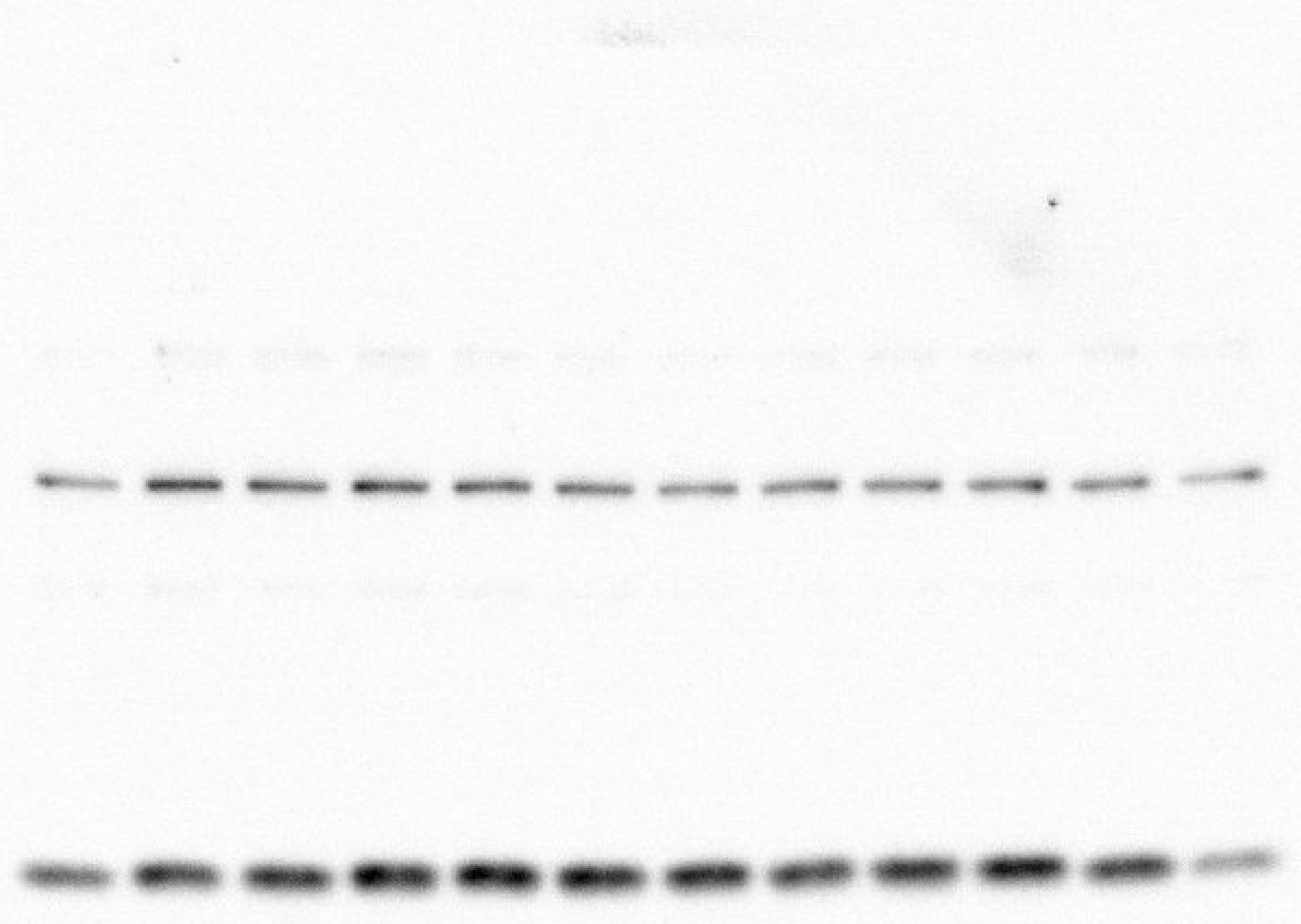

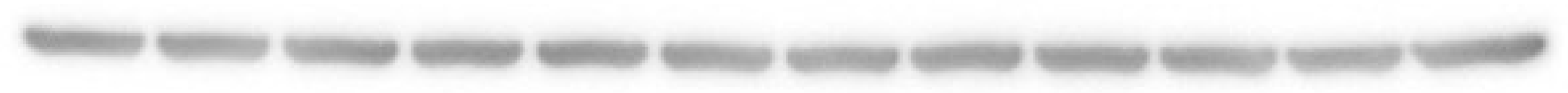

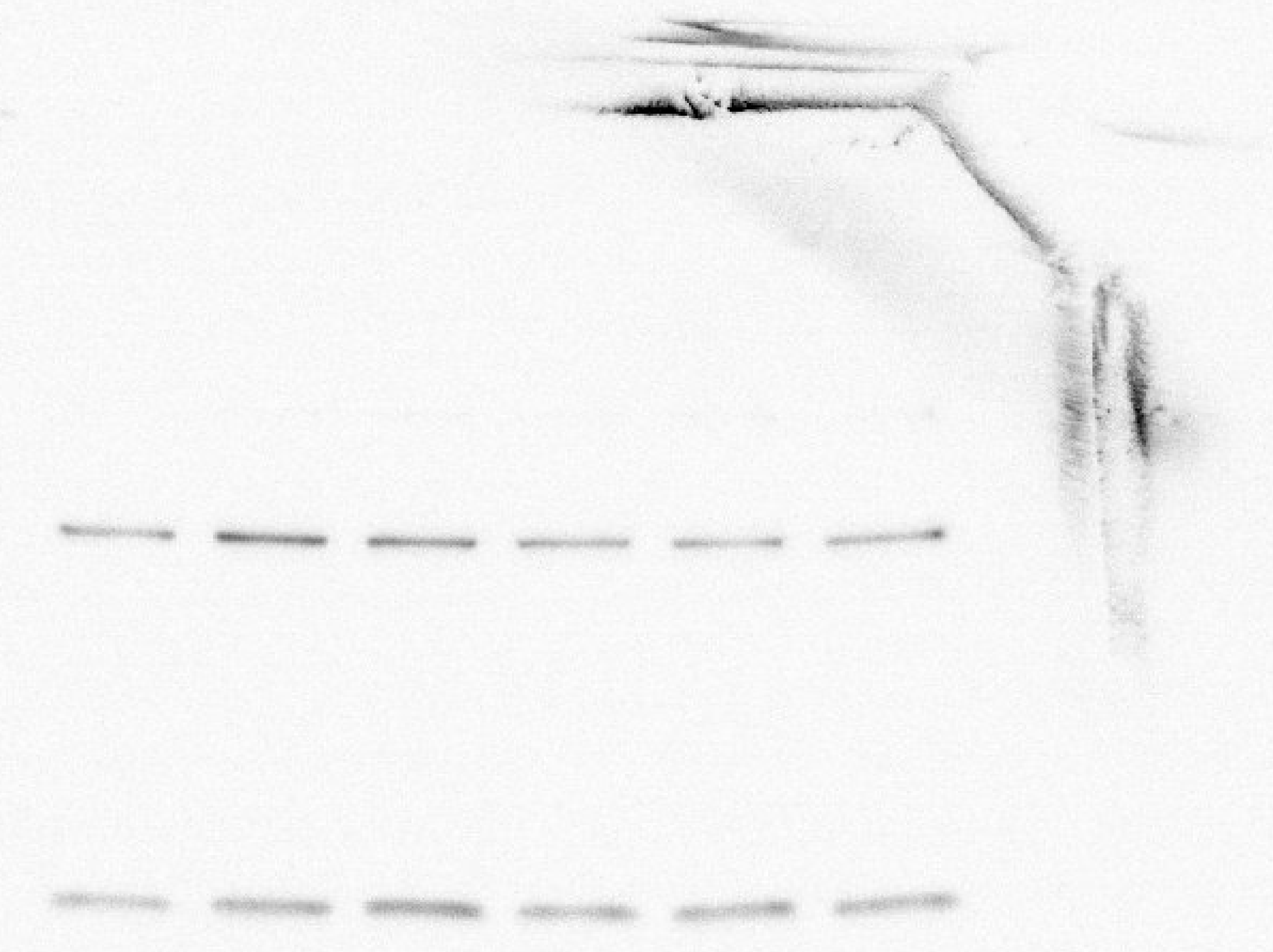

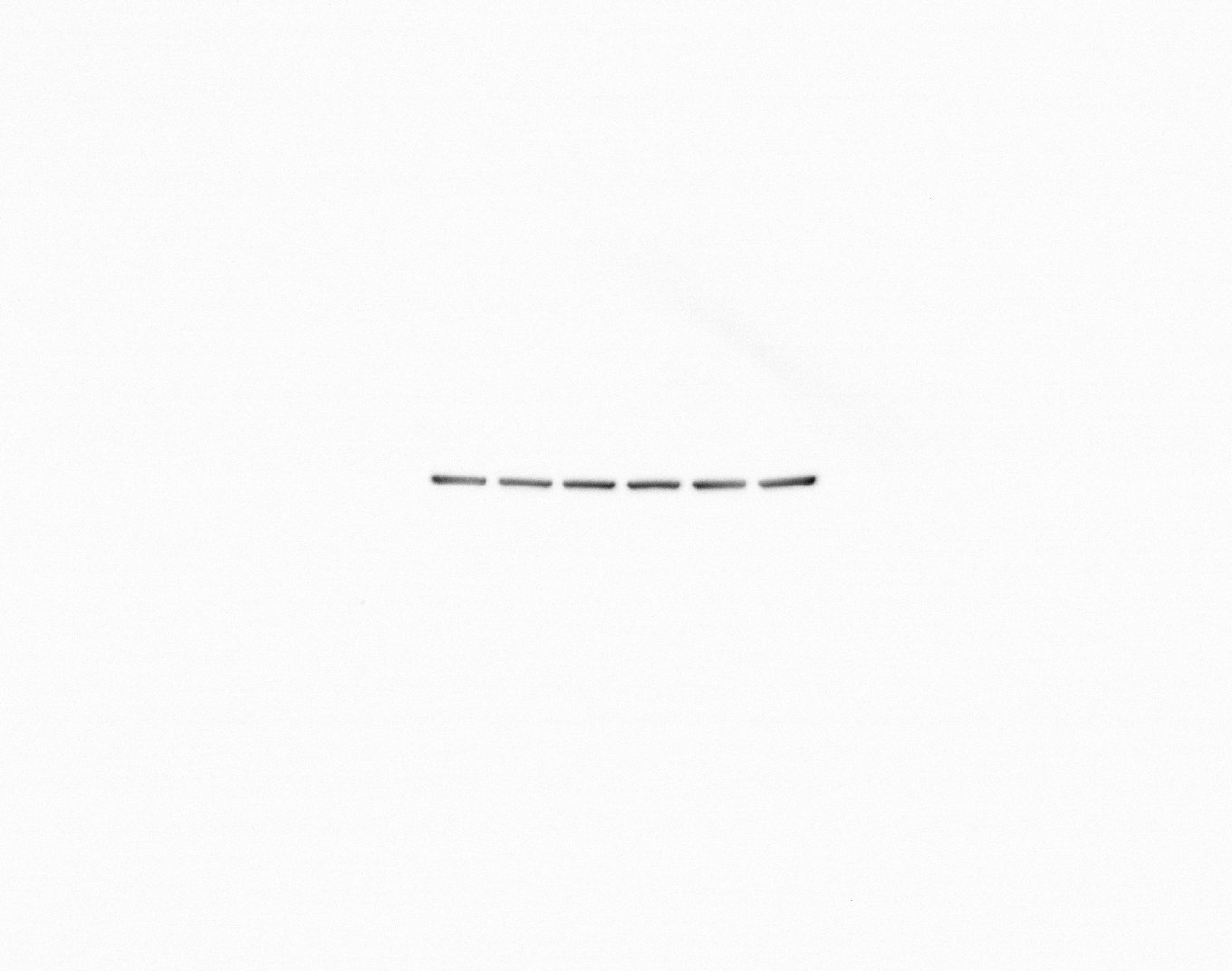

S1_raw_images (Fig. 7)

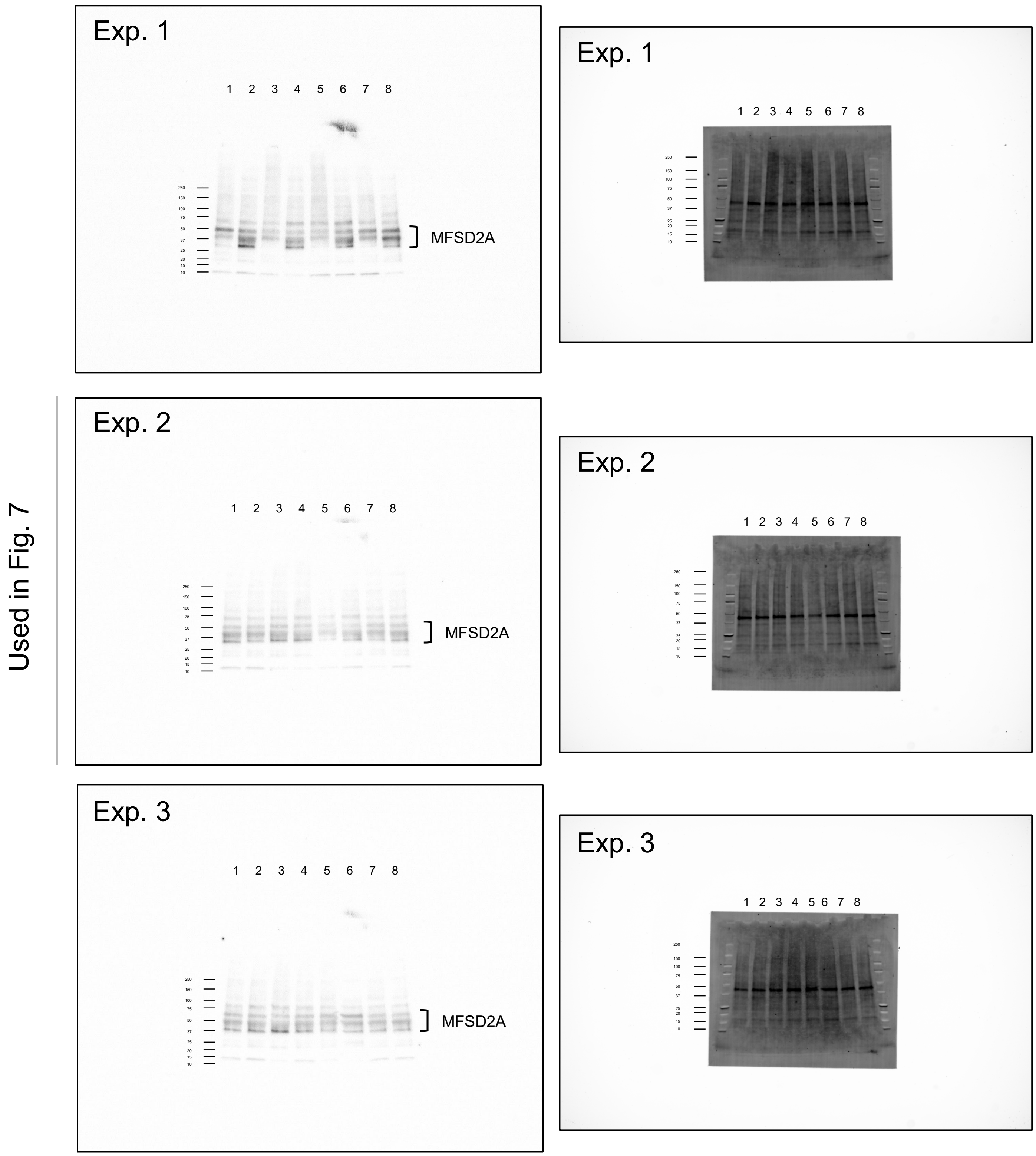

MFSD2A

Total protein (loading control)

Lane 1	2-month-old mice 1
Lane 2	2-month-old mice 2
Lane 3	8-month-old mice 1
Lane 4	8-month-old mice 2
Lane 5	12-month-old mice 1
Lane 6	12-month-old mice 2
Lane 7	24-month-old mice 1
Lane 8	24-month-old mice 2

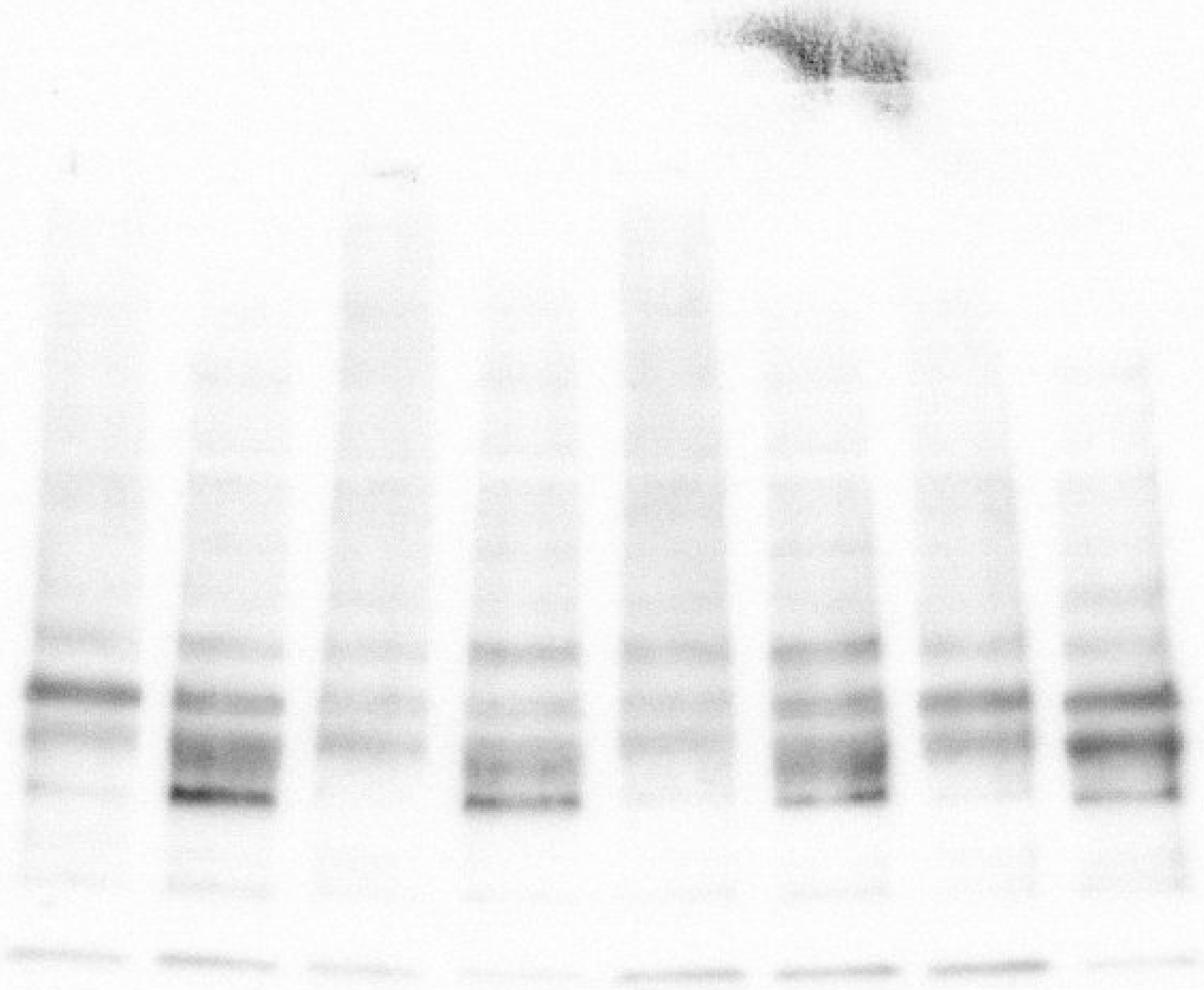

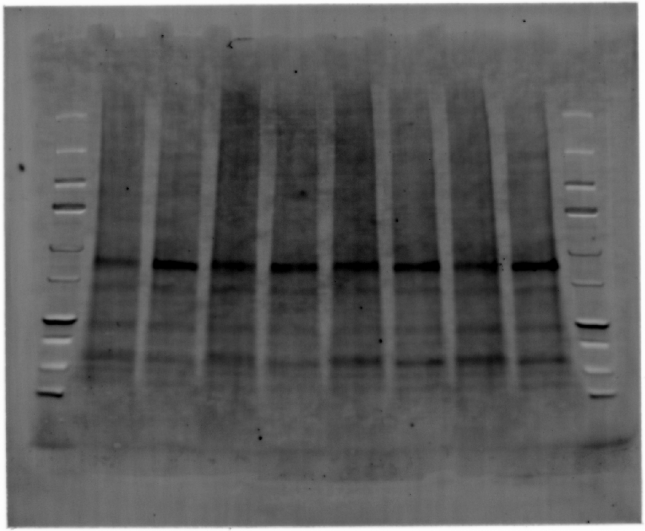

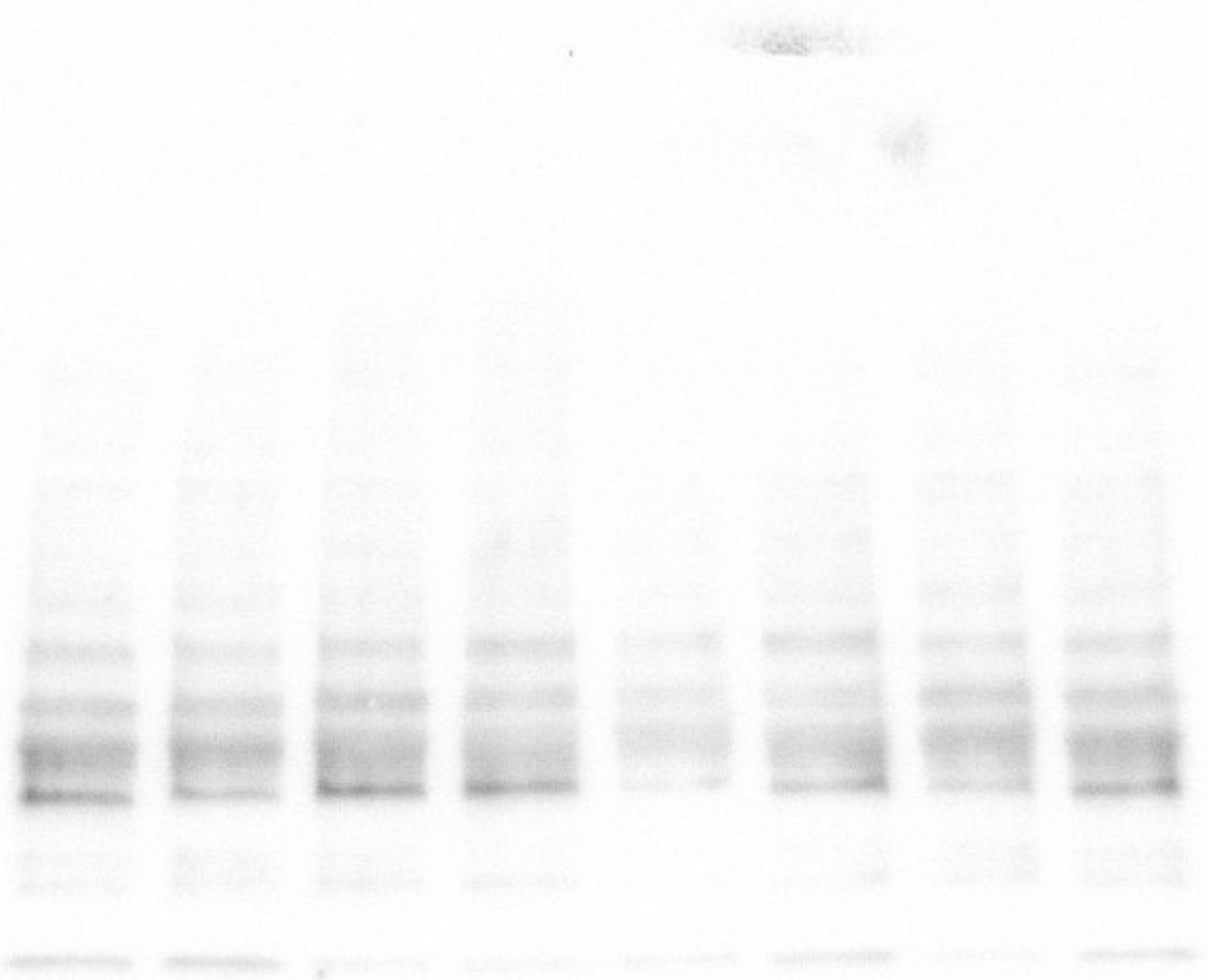

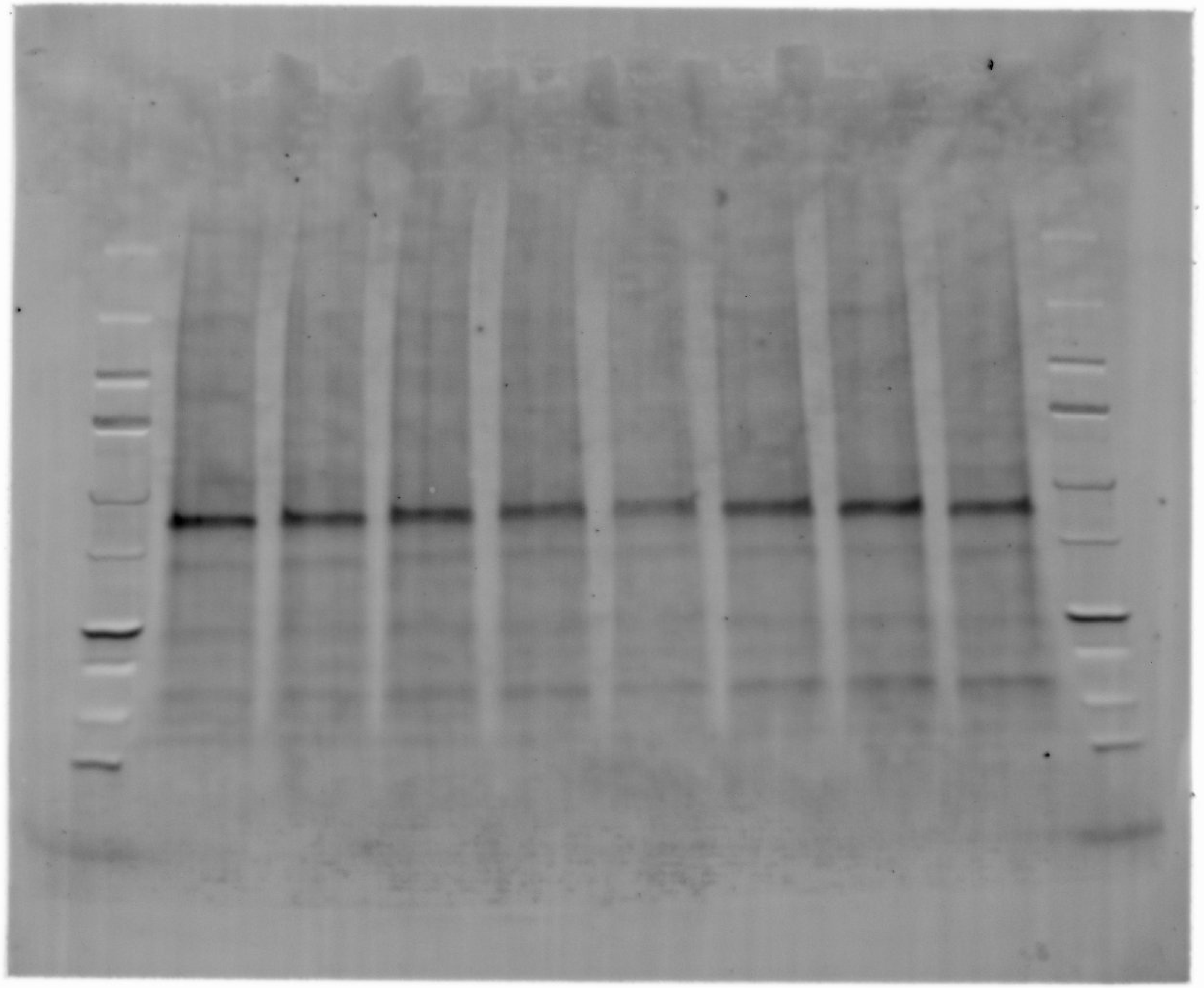

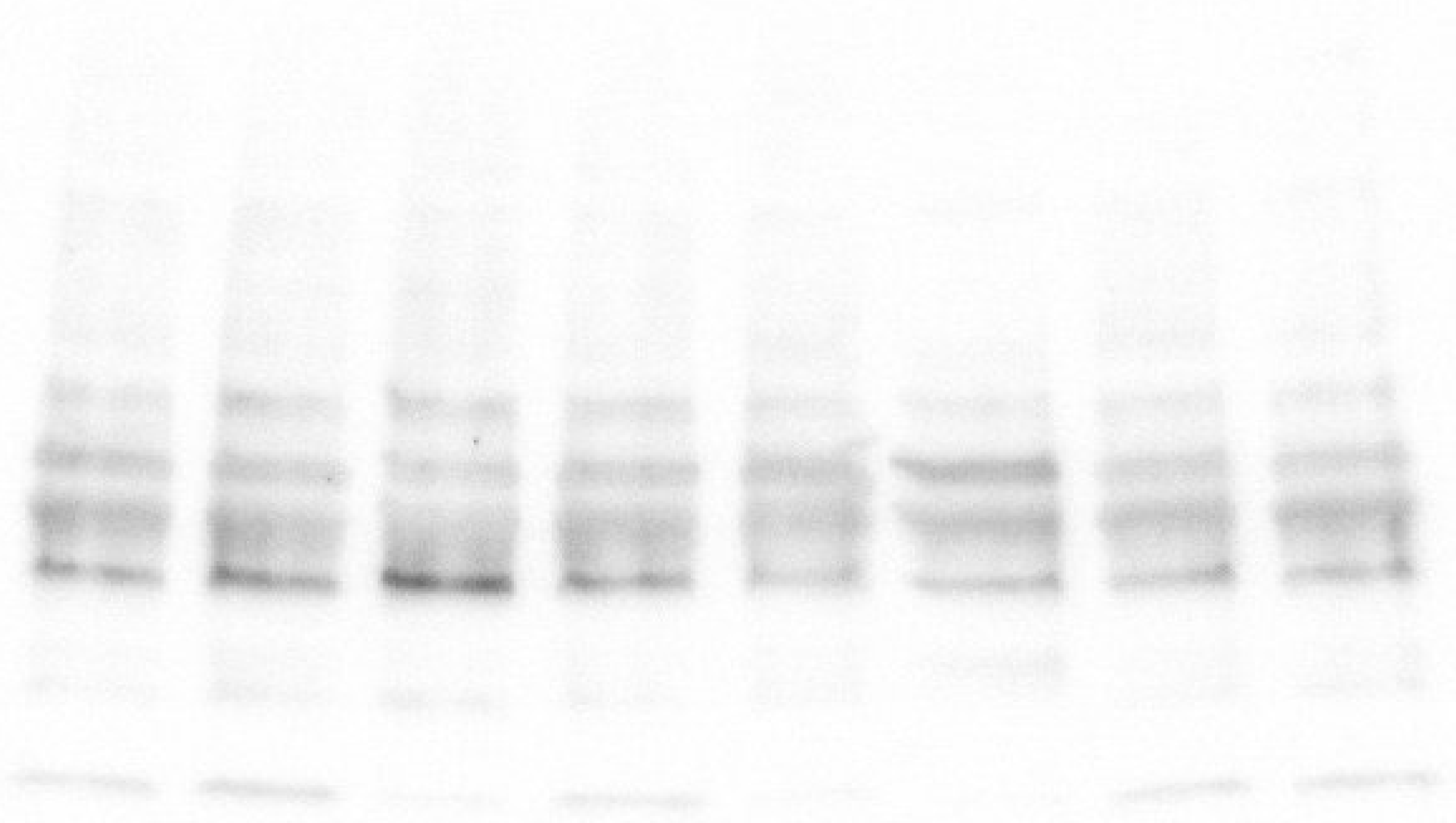

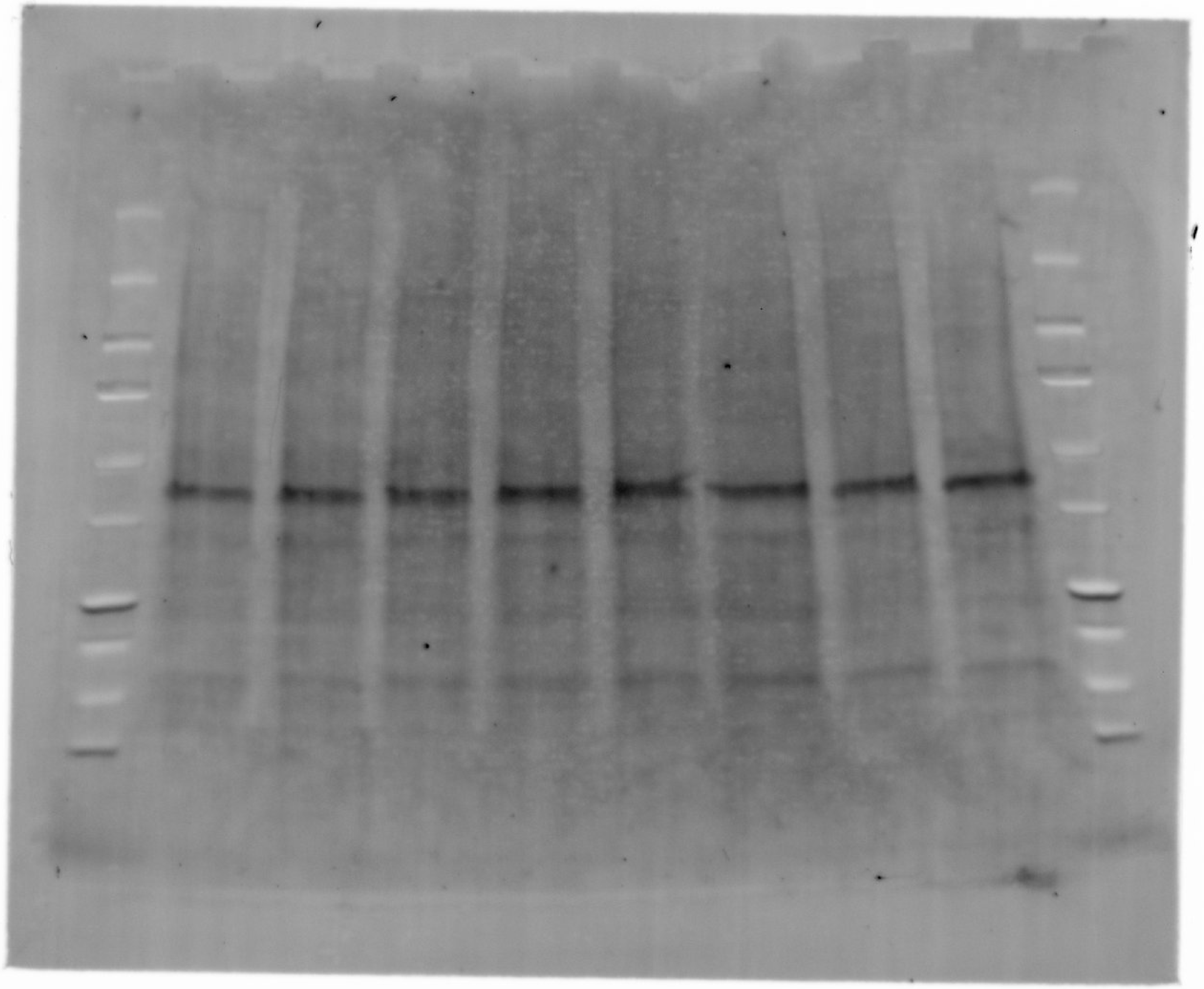

Supplement: S1 Raw images — (PDF) [file pone.0281946.s003.pdf]
